# Strengthening identification and characterization of causes of perinatal deaths in Kaski district of Nepal (Perinatal MITS Nepal)

**DOI:** 10.1186/s12884-025-07240-9

**Published:** 2025-02-04

**Authors:** Nuwadatta Subedi, Sunita Ranabhat, Sanjib Mani Regmi, Mukesh Mallik, Dela Singh, Shree Krishna Shrestha, Bandana Gurung, Arjun Bhattarai, Madan Prasad Baral, Sudhir Raman Parajuli, Ramchandra Bastola, Junu Shrestha, Sahisnuta Basnet, Eva Gauchan, Sabita Paudel

**Affiliations:** 1 Department of Forensic Medicine, Gandaki Medical College Teaching Hospital and Research Center, Pokhara, Gandaki Province Nepal; 2 Department of Pathology, Gandaki Medical College Teaching Hospital and Research Center, Pokhara, Nepal; 3 Department of Microbiology, Pokhara Academy of Health Sciences, Pokhara, Nepal; 4 Public Health Expert and Social Scientist Perinatal MITS Nepal, Pokhara, Nepal; 5 Department of Obstetrics and Gynaecology, Pokhara Academy of Health Sciences, Pokhara, Nepal; 6 Department of Pediatrics, Pokhara Academy of Health Sciences, Pokhara, Nepal; 7 Department of Obstetrics and Gynaecology, Gandaki Medical College Teaching Hospital and Research Center, Pokhara, Nepal; 8 Department of Paediatrics, Gandaki Medical College Teaching Hospital and Research Center, Pokhara, Nepal; 9 Department of Forensic Medicine, Pokhara Academy of Health Sciences, Pokhara, Nepal; 10 Department of Forensic Medicine, Manipal College of Medical Sciences, Pokhara, Nepal; 11 Department of Obstetrics and Gynaecology, Manipal College of Medical Sciences, Pokhara, Nepal; 12 Department of Pediatrics, Manipal College of Medical Sciences, Pokhara, Nepal; 13 Department of Pharmacology, Gandaki Medical College Teaching Hospital and Research Center, Pokhara, Nepal

**Keywords:** Cause of death, Death surveillance system, Minimally invasive tissue sampling, Stillbirth, Perinatal deaths, Verbal autopsy

## Abstract

**Background:**

Three million babies die in the early neonatal period while 2.6 million are stillborn per year worldwide, and one of three deaths can be prevented. The perinatal mortality rate in Nepal is around 31 per 1000 births. Although the perinatal and neonatal death rates have decreased recently, it still poses a major challenge to the health system of Nepal. The objective of the study is to determine the causes of perinatal deaths by integrating Minimally Invasive Tissue Sampling (MITS) in hospital perinatal deaths and incorporating verbal autopsy in community deaths in Kaski district of Nepal.

**Methods:**

The study will be conducted among the perinatal deaths reported in the five hospitals implementing the Maternal and Perinatal Death Surveillance and Response (MPDSR) system in Kaski district of Nepal. We will also conduct verbal autopsy (VA) among community perinatal deaths reported in the district. All the perinatal deaths reported in the study sites will be enrolled in the first stage of the study. Minimally Invasive Tissue Sampling (MITS) will be conducted among the consenting cases of perinatal deaths to retrieve relevant tissue samples and specimens. The specimens will undergo standard histopathological, microbiological, biochemical, and molecular tests. The “Cause of Death Panel” will finalize MITS informed cause of death following the customized protocol for the project and the cause so derived will be compared with that obtained by the review of deaths by the MPDSR committees of the hospitals. The Female Community Health Volunteers will be trained and mobilized to identify community perinatal deaths and trained personnel will conduct VA. Community engagement activities will be conducted to provide awareness to prevent perinatal deaths.

**Discussion:**

The mechanism of counting and accounting for deaths in a systematic manner is important and it can provide evidence to determine changes in clinical practice and to develop guidelines and training packages for preventive measures. The outcome will be helpful to standardize methods to establish the accurate causes of perinatal deaths and develop strategies to minimize the deaths. The selected pathological investigations can be integrated into the existing death surveillance system in order to effectively determine the causes of death.

## Background

Globally, neonatal deaths account for approximately 47% of the total child deaths under the age of five years. The mortality of under-five children is decreasing globally and the deaths in this age group are more concentrated in the initial days of their lives. In 2020, approximately 2.4 million children died in the first month of their lives amounting to 6500 neonatal deaths every day. One-third of these deaths occur within the first day and nearly three-fourths of them die within the first seven days after birth [1]. One in three of these fatalities can primarily be avoided [2]. In addition, stillbirth is also a global burden with an estimated 1.9 million babies stillborn at 28 weeks of pregnancy or later in 2021, amounting to a global stillbirth rate of 13.9 stillbirths per 1,000 total births. The globally represented number of stillbirths is mostly concentrated in developing countries and is highest in sub-Saharan Africa and Southern Asia [3]. In some parts of developing nations, childbirth is frequently viewed as a natural procedure that does not require preparation or medical attention and often in rural areas, childbirth occurs at home without skilled birth attendant and medical care [4]. Though the situation has lately improved, efficient implementation of better healthcare services can significantly reduce stillbirths and newborn fatalities in low- and middle-income countries (LMICs) [5].

Perinatal mortality is defined as the fetal deaths past 28 completed weeks of pregnancy and deaths among live-born children up to seven completed days of life [6]. Out of four million global perinatal deaths, approximately 98% occur in developing countries including Nepal [7]. The perinatal mortality in Nepal was 31 per 1000 total births as reported in 2016 and the figure is quite improved as compared to data from 2006 when the rate was 45 per 1000 total births [8]. Despite recent declines, maternal and neonatal mortality rates remain a significant problem for Nepal’s healthcare system. The first step in addressing this issue is to assemble baseline data, quantifying such deaths and determining their causes. This will assist in starting the process of developing a solution plan, determining whether these solutions are effective, and finally assisting in the formation of pertinent guidelines.

Perinatal deaths are reviewed under the program, Maternal and Perinatal Death Surveillance and Response (MPDSR) System in Nepal. MPDSR is a type of continuous surveillance method that connects local and national health information systems and quality improvement processes. It entails the routine identification, notification, quantification, and determination of all maternal and perinatal fatalities, as well as the utilization of this information to respond with steps that will prevent future deaths. It collects data on preventable factors that cause maternal and perinatal deaths and uses it to guide actions that must be taken at the community level, within the formal health-care system, and at the inter-sectorial level (i.e., in other government and social sectors) to prevent similar deaths in the future. The occurrence of perinatal deaths reported in the health facilities is investigated, and the attending healthcare provider fills out the Perinatal Death Review (PDR) form. The hospital MPDSR committee meeting is conducted once a month to identify the cause(s) and leading factors of death along with recommendations for actions and implementation of further actions. The information on maternal conditions in perinatal deaths is analyzed minutely, and the demographic and clinical information is systematically reviewed and analyzed to establish Cause of Death (CoD), potential maternal conditions, preventability, and future actions [9].

The determination of CoD in perinatal deaths is an important aspect of the perinatal deaths review system. Among 145 perinatal deaths investigated in a MPDSR implementing hospital in Nepal, intrapartum hypoxia, prematurity, and premature labor were identified as the common causes [10]. However, ascertainment of the CoD in such reviews is based on clinical and demographic information only. The sparse availability of investigations and the non-performance of an autopsy can make it difficult to determine the cause of death in stillbirths. A complete diagnostic autopsy (CDA) should be performed to overcome this and the CDA should be supplemented with ancillary investigations. However, there are several barriers to CDA, such as cultural and religious beliefs, delays in the funeral process, unavailability of resources, and a shortage of trained experts. To investigate CoD in various age groups, minimally invasive tissue sampling (MITS) has recently been employed successfully in many countries, and it has been particularly helpful in perinatal and child mortality [11, 12]. The correct CoD is frequently overlooked in Nepal since CDA is seldom carried out unless there is a medico-legal reason, and this can be overcome by the use of MITS. MITS is a relatively non-invasive process that may be carried out in hospitals or even in the community by trained pathologists or technicians. It is practical in our situation and can be very helpful in determining the cause of death in perinatal deaths [12]. Evidence suggests that MITS is a procedure that is acceptable in contexts with diverse cultures and religious beliefs since it is less disfiguring and takes less time than CDA, which prevents delays for funerals [13, 14]. A consent rate of 71% has been presented from a study involving under-5 mortality and stillbirths, at five sites in Mozambique, South Africa, Kenya, Mali, and Bangladesh [15]. MITS has already been successfully implemented in the adult population and was useful in identifying the cause of death in most cases and has been identified to be a valuable tool in our setting [16, 17]. The use of MITS has been expanding globally and it has been proven to be effective in the determination of CoD. In comparison to CDA, MITS is a viable and comparable alternative method for postmortem evaluation [11].

In resource-constrained environments where many deaths occur outside of hospital facilities and certification of the reasons for death is frequently unavailable, verbal autopsy (VA) can be another technique for determining the causes of death. In the absence of clinical examinations and CDA, VA provides a partial answer to determine the reasons for death. A VA is a process used to identify the cause of death based on a structured conversation with the deceased’s caretakers or family members who had enough knowledge of the deceased’s conditions. It can be used in communities having limited or no access to hospitals and medical certification of CoD. A systematic questionnaire is used for this, which investigates the medical details, signs, symptoms, and circumstances that lead to death. The WHO has standardized the VA tool to maintain the standards of ascertaining the causes of death [18] and the tool has been proven to identify the main causes of neonatal deaths in India [19] and Pakistan [20]. The tool has also been used in some districts of Nepal to identify the causes of perinatal deaths [21–23]. Since 1956 when WHO first showed its interest in VA, many changes have been made in the instrument. In 2016, WHO VA was updated for the automated interpretation of VA data through the implementation of software programs. Similarly, the 2022 VA instrument includes questions for ascertaining death due to COVID-19 [24].

The MPDSR system is an effective Death Surveillance System (DSS) conducted with the objectives of identifying the cause of death and preventive measures to minimize maternal and perinatal deaths. In Nepal, the program is based on a review of the demographic and clinical findings alone in perinatal deaths and VA in addition in maternal deaths [9]. Autopsy and additional laboratory investigations are not conducted, so many causes of death could have been missed, which can be overcome by incorporating MITS. This can also be useful to strengthen the current DSS and advocate for the incorporation of MITS in the DSS in the future. The MPDSR system investigates hospital perinatal deaths only and community perinatal deaths are neither reported nor registered in the Vital Event Registration System (VERS). Performing VA in community perinatal deaths can provide the causes of deaths and provide information related to such deaths. The study is planned to determine the causes of perinatal deaths by integrating MITS in hospital perinatal deaths and incorporating verbal autopsy in community deaths in the Kaski district of Nepal.

The specific objectives of the study include:

1. To determine the primary causes of perinatal deaths.
2. To determine the causes of death in the causal chain of events and contributory causes associated with perinatal deaths.
3. To compare the MITS informed cause of death with that of assigned by the existing DSS.
4. To categorize causes of death and the specific pathogens associated when infection is the cause.
5. To perform verbal autopsy to find the cause of death among community perinatal deaths.
6. To explore the maternal and obstetric risk factors for perinatal deaths.
7. To explore the potential preventability of perinatal deaths.
8. To find the association of perinatal death characteristics and acceptance of MITS in the study population.

## Methods/Design

All the perinatal deaths reviewed under the MPDSR system in the Kaski district of Nepal will be the target population. The MPDSR system covers all the perinatal deaths reported in the hospitals selected by the Family Welfare Division of the Department of Health Services of the Ministry of Health and Population (MoHP). Five hospitals are implementing the MPDSR system in the district. They are: Gandaki Medical College, Pokhara Academy of Health Sciences, Manipal College of Medical Sciences, Matri Shishu Miteri Hospital, and Shishuwa Hospital. The project will include all the hospitals to make the sample representative of the district as much as possible. Kaski district lodges major referral hospitals of the province and the only three medical colleges in the province are in this district. So, most of the cases are referred to the study centers from the other districts of Gandaki province.

The perinatal deaths reviewed under the DSS include late fetal deaths and early neonatal deaths (death within 7 days of birth) more than or equal to 28 weeks’ gestation or weighing 1000 g and above. If the last menstrual period (LMP) or gestational week is confirmed, the weeks of gestation are considered, whereas if the gestational age is not sure or not known, the birth weight of the fetus is considered and if birth weight is also not known, then the length of (more than 35 cm) of the fetus is considered to classify as perinatal death. When the LMP is unknown and the first trimester ultrasound is available, it will be used to calculate the gestational age [25].

All the perinatal deaths falling under the criteria of MPDSR will be approached for enrollment in the first phase of the study. The nearest relatives of the deceased will be counseled by the treating clinical team and after the regular counseling is completed, trained healthcare professionals will approach the relatives of the deceased for informed consent to enroll in the study. We have prepared a standard operating protocol for the approach to the family members and the consenting procedure. The professionals involved in the informed consent process will be initially trained and periodic refresher sessions will also be conducted. The nearest family members of the deceased will be informed of the purpose of the study and its importance in the determination of the causes of death and its integration with the existing death review system. The family members will be first counseled for MITS. If consent for MITS is not obtained, they will be counseled for the examination of the placenta if available, and for the clinical and demographic data. The acceptance and denial of the MITS procedure, placenta examination, and clinical and demographic information will be analyzed concerning the characteristics of perinatal deaths.

The sampling unit for hospital perinatal deaths consists of perinatal deaths reported in the study sites and for verbal autopsy it is the community perinatal death reported in the Kaski district of Nepal.

### Sample size and sampling technique

The study will be of three years duration starting from March 2023. In the first phase, we will enroll all the available hospital perinatal deaths in the study duration. We will continue to enroll cases in MITS till we get a sample size of 300 or three years from the commencement of the study. For verbal autopsy: As the community perinatal deaths are not formally reported or registered into the VERS, the exact number is not known. However, we will enroll all the notified and consented cases during the study period. This will be a census study enrolling all the eligible perinatal deaths reported in the study sites during the study duration.

### Inclusion and exclusion criteria

All the perinatal deaths reviewed under the MPDSR system in the hospitals implementing the program in the Kaski district of Nepal will be the target population. As the PDR is a mandatory part of MPDSR, all the perinatal deaths are investigated under the system, however, a separate consent will be taken for clinical data abstraction for the study purpose. We will also approach for the consent for placental examination, when available. To enroll the cases into MITS, we will include the perinatal deaths within 24 h of death and up to 72 h if the body is refrigerated shortly after death. It has been demonstrated that the tissue samples for diagnostic tests to determine the cause of death can be successfully collected later than 24 h [26] and even up to 72 h after death [27]. Likewise, to enroll a perinatal death into the VA, we will include all the community perinatal deaths reported in the Kaski district during the study period, when the information is obtained within one year of such deaths.

The grade of maceration will be evaluated by the MITS experts and the cases with Grade III maceration will be excluded for MITS. Likewise, the neonatal deaths that are reported after 24 h of death and not preserved in the cooling chamber and with evidence of decomposition will be excluded. The maceration of stillborn fetuses is graded as 0 to III as represented in Table 1 [28]. The cases which are excluded for MITS will be approached for placental examination, when available and also for the clinical and demographic information.

**Table 1:** Classification of the grade of maceration

Grade	Time since death	Characteristics
Grade 0	< 8 h	“Parboiled” skin discoloration
Grade I	≥ 8 h	Not specified desquamation of the skin
Grade II	2–7 days	Blood-stained effusions in serous cavities and skin peeling
Grade III	≥ 8 days	Yellow-brown liver, turbid effusions and mummification

The exclusion criteria for VA include the hospital-based perinatal deaths and reviews done by the MPDSR system and the deaths that had occurred more than one year earlier than the reporting date.

### Data collection technique

A structured interview will be conducted with the nearest relative of the deceased baby using the case report forms (CRF) prepared for the study. We will use CRFs for data collection under different categories and they include eligibility, consent process, maternal medical and birth clinical history, stillbirth evaluation, pediatric/neonatal clinical history, MITS specimen collection, placenta collection, MITS gross examination, pathology report, laboratory results, and certificate of cause of death forms. The project-specific health care professional designated to counsel the relatives and take the informed consent will be scheduled for their prior duty and will attend the cases throughout the duration of 24 h and all days of the week after getting death notification from the concerned departments of the study sites. We will also review the clinical notes of the case for the duration of the hospital stay if the case is treated in the hospital. The vitals, clinical diagnosis, medications or procedures performed and progress during the hospital stay will be noted. Any investigation reports of both the mother and the deceased baby, if available will also be used to complete the CRFs. The trained MITS experts will be on duty throughout the 24 h on all days of the week and will attend the call from the respective hospitals soon after the call as per their duty. The MITS procedure will also be conducted as soon as possible in the designated MITS rooms beside the maternity wards and/or neonatal intensive care units of each hospital. The overall MITS process will take about 30 min to one hour.

### Examination of the deceased and MITS procedure

After obtaining informed consent from the nearest relatives of the deceased, we will perform meticulous physical examination, and anthropometric measurements, take photographs, and perform MITS in an established protocol [29]. The MITS expert; a forensic pathologist trained to perform MITS, on duty, will perform the examinations and collect samples whereas a trained MITS assistant will complete the CRFs, take photographs, and assist the expert in conducting MITS.

### Anthropometric measurements

#### Weight (in grams)

The body will be placed on a scale and the weight will be measured.

#### Length

The body will be placed lying on the side with legs extended and, using the flexible tape, the distance from the vertex (top of head) to the heel of the right foot will be measured.

#### Head circumference

The measuring tape will be placed around the head so that the tape lies across the frontal bones of the skull, slightly above the eyebrows, perpendicular to the long axis of the face, above the ears, and over the occipital prominence at the back of the head to obtain the head circumference.

#### Lower leg length

The measurement of the right leg from the medial malleolus to the medial condyle of the tibia will be recorded.

#### Foot length

The distance from the heel to the longest toe of the right foot parallel to the long axis of the foot will be recorded.

#### Body inspection

We will inspect the external genitals to record the sex. It will be determined whether the body is fresh or macerated (skin and soft tissue changes such as skin discoloration or darkening, redness, peeling, and breakdown), and in case of maceration, the grade will be evaluated. A detailed external inspection of the whole body will be done looking for visible congenital physical anomalies or malformations, evidence of trauma, external tumors, skin rashes, and lesions, or changes in the color of the skin (e.g. depigmentation, areas of darkness).

#### Body cleaning and sterilization

The bodies will be initially washed with clean tap water. The areas of the body to be punctured for MITS will be disinfected with 96% alcohol and iodine solution, allowing five minutes for each to act on the surface of the body for adequate disinfection for the microbiological analyses and the skin will be allowed to air dry.

#### Postmortem collection of specimens using MITS

The nasopharyngeal sample will be collected by sterile swab in BHI broth. Another nasopharyngeal swab will be collected in Viral Transfer Media (VTM). We will collect 3 to 5 mL cerebrospinal fluid (CSF) using a 20G spinal puncture needle by occipital approach, 5 to 10 mL of blood by 18G intramuscular needle, and a 20 mL syringe using the supraclavicular approach or direct puncture to the heart, brain tissue samples for histology, lung tissue samples for microbiology and histology and liver tissue sample for histology using MITS with the sterile Bard Monopty needles. Separate samples will be taken from the right and left lungs and collected in VTM.

#### Laboratory Investigation

The CSF samples will be examined microscopically by Gram stain. They will be further cultured on MacConkey agar, blood agar, and chocolate agar plates and incubated at 35 °C for seven days. Chocolate agar plate will be incubated at 35 °C having an environment of 5 to 10% CO2. A 3 mL sample of blood will be kept in (Brain Heart Infusion) BHI broth and incubated for seven days. The broth will be checked for any growth or turbidity and further subcultured in Blood agar and MacConkey agar plate. The nasopharyngeal sample collected by sterile swab will be inoculated in BHI broth for bacterial culture and incubated at 35 °C for seven days. If there is any growth or turbidity, it will be further subcultured in Blood agar and MacConkey agar plate. Bacterial isolates will be identified based on their biochemical features, cell shape, gram stain reactivity, and cultural traits as per the description by Monica Cheesbrough [30]. Another nasopharyngeal swab collected in Viral Transfer Media (VTM) will be subjected to Real-Time Polymerase Chain Reaction (RT-PCR) for respiratory pathogens. The TRUPCR^®^ Respiratory Pathogen Panel Kit will be used an in vitro diagnostic test for the qualitative detection of microorganisms mentioned in Table 2 from the nasopharyngeal swab samples and bacterial/viral cultures using RT-PCR method [31]. Likewise, simplex PCR will be performed to detect an additional pathogen; Parvovirus B19. The right and left lung tissue samples will be taken and analyzed for bacterial agents. 3 mL of blood placed into Ethylenediamine tetraacetic acid (EDTA) tubes will be centrifuged to separate the plasma supernatant. Plasma samples will be tested for the presence of antibodies against human immunodeficiency virus (HIV) 1/2, hepatitis B virus (HBV) surface antigens, Treponemal antibody test, and malarial antigen test using commercially available rapid diagnostic kits. TORCH profile will be performed using commercial kits of CLIA, Maglumi. We will also perform tests for blood group and Rh typing. Using EDTA blood, thick and thin smears for malarial parasites, micro ESR, Absolute neutrophil count (ANC), and Immature to total cell ratio (IT ratio) will be studied.

The tissue specimens collected for histological analysis will be fixed with 10% neutral buffered formalin for at least four hours and then passed into a tissue processor for eight hours, embedded in paraffin, and cut into four-micron sections, which will be then stained with hematoxylin and eosin (H&E) as per standard procedures. The H&E sections of all tissues will be initially evaluated, and whenever necessary, ancillary histochemical tests i.e., Ziehl-Neelsen and Periodic Acid Schiff will also be performed. All the cores of tissues collected in each block will be carefully evaluated. Examination and recording of all the slides will be done under a microscope Olympus CX 23, in 10X, 40X, and 100 X magnifications. Karyotyping will be done in suspected or known congenital anomalies. Any further laboratory tests will be performed on a need basis and the type of case. The summary of laboratory tests is presented in Table 3.

**Table 2:** Pathogens to be detected by PCR through respiratory panel

Viruses	Bacteria
1. Human Parechovirus 2. Human corona virus (alpha & beta) 3. Human parainfluenza virus 1–4 4. Influenza A virus 5. Pandemic H1N1 influenza virus (PDM H1N1) 6. Influenza A (H3N2) virus, 7. Influenza B virus 8. Influenza C virus 9. Enterovirus 10. Human metapneumoviruses (A/B) 11. Human adenovirus 12. Human respiratory syncytial viruses (A/B) 13. Human rhinovirus 14. Human bocavirus	1. Staphylococcus aureus 2. Streptococcus pneumoniae 3. Klebsiella pneumoniae 4. Mycoplasma pneumoniae 5. Salmonella spp. 6. Streptococcus pyogenes 7. Bordetella spp. 8. Chlamydia pneumoniae 9. Streptococcus agalactiae 10. Acinetobacter baumannii 11. Pseudomonas aeruginosa 12. Legionella pneumophila, 13. Haemophilus influenzae (A-F) 14. Moraxella catarrhalis

**Table 3:** Summary of the laboratory diagnostic tests*

Specimens	Diagnostic tests
Nasopharyngeal swab (in early neonatal deaths only)	• Gram stain and bacterial culture • PCR
Placenta	• Histology • Microbiology (bacterial culture)
Blood	• Bacterial culture • Serology (HIV, Treponemal antibody test, HBsAg) • TORCH screening (toxoplasmosis, rubella cytomegalovirus and herpes simplex virus 2) • Blood grouping & Rh typing • Bilirubin • Malarial antigen test, thick and thin smear for blood parasites • ANC (Absolute neutrophil count), • IT ratio (Immature to total cell ratio) • Karyotyping in known or suspected congenital anomalies
CSF	• Microbiology (Culture, Gram stain) • CSF analysis
Brain	• Histology
Lungs (right and left)	• Histology • Microbiology (bacterial culture)
Liver	• Histology

*This is not an exhaustive list. Additional lab tests performed during the lifetime of the neonate and the mother can also be used to establish the diagnoses

### Verbal autopsy in community perinatal deaths

#### VA tool

VA is a technique to collect information through a valid and well-structured set of questions to ascertain the cause of death. For this study, the 2022 WHO VA questionnaire will be used which is suitable for all age groups. It has three forms, whereas we will be using WHO VA questionnaire-1 which is designed for neonatal deaths, perinatal deaths, and stillbirths (deaths of children aged below four weeks). The 2022 WHO VA instrument and other supporting documents for implementation are available from the website [24]. Questions will be translated into the Nepali language as per the guidelines of the 2022 WHO VA so that they can be used suitably and effectively in the local set-up.

#### Approach to respondents for VA

VA is done for community deaths when hospital certification is not available. Therefore, sensitization of the community, identification of suitable respondents, and selection of the appropriate time are the key factors. We will involve community leaders and female community health volunteers (FCHVs) to list out the incidences of deaths along with their contact addresses and inform the households of the importance and methods of the VA procedures. We will collect data after two weeks and within 12 months after the death occurrence to decrease the recall bias. As a respondent, it has been recognized that the mother is almost the best respondent. Thus, we will interview the mother even if we need to make the next visit. If it would be impossible to find the mother, then we will approach the eldest member of the family who can provide the information related to the death.

Nursing staff will be trained to interview the VA with empathy, the issue being sensitive. They will read out the consent form and receive the written consent before collecting the data. The mode of data collection can be paper-based, or software-based. We will primarily use software programs on tablets for the data collection where feasible and use paper forms when electronic methods may not be feasible considering geographical and technological barriers. Data collected electronically can be interpreted automatically by computer algorithms to predict the most likely cause of death. WHO has recommended three analytical tools for the cause of death interpretation amongst which we plan to use open VA. When a software-based questionnaire cannot be used, we will use a paper-based approach. We will send the completed questionnaire to the physicians for review who use diagnostic guidelines and their clinical judgments for ascertaining the cause of death.

### Assignment of cause of death

#### Cause of death without MITS

The MPDSR system mandates filling the PDR form by the attending health care provider and assigning the CoD in all cases of perinatal deaths in a predefined format. The deaths are also reviewed by the MPDR committee in scheduled monthly meetings, and the CoD can be further revised based on the discussions and suggestions of the committee members The primary CoD is identified and coded as per the guidelines of the WHO application of ICD-10 to deaths during the perinatal period (ICD-PM) [32]. The committee members involved in the review of cases will be blinded by the results of MITS findings till MITS informed CoD is established.

#### MITS informed cause of death

In cases undergoing MITS, we will prepare a summary of case details using a standard format specifically prepared for the project including the demographic details, details of the mother, clinical details of the baby, examination findings of the deceased baby and laboratory examination from MITS samples. The case and institution identifiers will be removed, and a unique identifier will be allocated by the research coordinator. A CoD panel involving a coordinator, pathologist, microbiologist, obstetrician, and pediatrician with expertise in neonatology will be constituted to review the case report forms. The CoD ascertained by the MPDSR committee of the case will be blinded to the panel members and none of the members of the panel will review the cases that they had participated in reviewing the cases during routine MPDR meetings. This will be maintained by engaging the members from other hospitals than where the death had occurred or those who were not on the review committee of MPDSR for the particular case, or their involvement will be accepted only after the washout period of at least two months after the review. The primary cause of perinatal death will be identified by the panel. The attribution of the CoD will be done as per the guidelines of ICD-PM [32], and we will also establish the causal chain of events from the CoD so obtained. A guideline for the diagnostic standards has been prepared based on that used for the Determination of the Cause of Death (DeCoDe) process by CHAMPS [33] and PURPOSe study [34, 35].

#### Determining infection as a cause of death

The detection of microorganisms alone will not be regarded as the cause of perinatal deaths. For considering infection as the cause, the PCR and/or culture results will be correlated with the clinical findings of infection. We will also consider CRP, Differential Leukocyte Count (DLC), and chest X-ray findings to consider infection in the causal chain of events.

#### Cause of death from VA

The completed VA forms, if taken on paper, will be provided to two trained pediatricians with experience in neonatal care working as faculty members who will determine the CoD based on consensus. The DeCoDe diagnostic standard will be used for the ascertainment of the cause of death.

The classification of CoD will be done according to the guidelines of ICD-PM based on the classification of Antepartum, Intrapartum, and Neonatal deaths. Maternal conditions responsible for the perinatal death will also be classified accordingly.

#### Potential preventability of perinatal deaths and recommendations for action

We have developed project-specific guidelines to identify potential preventability of the perinatal deaths. Potentially preventable conditions will be defined as the conditions or diseases which could have been identified if taken to the health care facility on time and if identified, the condition could be prevented with existing treatments in the facility. Whereas the condition will be regarded as not preventable if the diseases and conditions are sufficient in the ordinary course of nature to cause death and non-treatable condition under the circumstances. The potential preventability of perinatal deaths will be identified by the consensus of the DeCoDe panel members. The committee will also provide recommendations for action to prevent similar deaths in the future in a structured format.

Once the CoD is ascertained by the panel and the form for the preventability of perinatal deaths and recommendations for action are completed, the research coordinator will incorporate the case identifiers in the case summary, and it will be provided to the concerned departments of the study sides from where the case was enrolled. The designated clinician will review the summary and will provide recommendations for action for individual cases. The aggregate recommendations will also be shared with the government offices responsible for the implementation and monitoring of the MPDSR program.

After we remove all identifiers, we will securely store, use, and share these data for future research. We will store the data in a central archive ensuring that the personal identifiers will not be included in such archive. These data can be made available for other research studies that may be done in the future after a certain embargo period. This could be research about the CoD or other diseases and conditions to better understand and prevent early death. The name and identifying information will be removed from any data provided before they are shared with other researchers. The link between the name of the deceased and their data will be maintained and secure at Gandaki Medical College and Research Center, Pokhara.

#### Data analysis and statistical tools

The data will be collected in the REDCap system developed for the study. The data will be exported to STATA 15.1 for further analysis. Concordance between the MITS diagnosis and the clinical diagnosis will be calculated under different categories of causality by using Cohen Kappa. We will explore the level of agreement between the two methods in excess of agreement due to chance statistically. Our assumption is that if MITS is integrated into the DSS, a better outcome will be achieved due to the synergistic effect. Therefore, we will also perform regression analysis with effect modification so that any interaction between exposure can be reported. We will also find the association between variables using univariate analysis and multivariate multinomial logistic regression analysis with effect modification to report the interaction between variables. The p-value of < 0.05 will be considered significant.

## Discussion

We can identify the causes of death by incorporating MITS into the perinatal death surveillance system so that effective preventive strategies can be adopted. The prevention strategies can be implemented by different stakeholders as per their scope. The potential strategies adopted at the hospital level could be the upgradation of infrastructure and resources, infection control measures, quality control, monitoring and regulation, etc. The departments also can act in quality control and infection control measures. The media can be informed accordingly on the potential implications. The other potential stakeholder is the public health office which can implement various programs in creating awareness among healthcare professionals as well as community members. This can also be useful to strengthen the MPDSR system and advocate for the incorporation of MITS in routine cases in the future. Performing verbal autopsy in community perinatal deaths can explore the status of such deaths as they are not reported elsewhere. We can find out the causes of those deaths and can generate evidence for planning and preventive purposes.

Every Newborn Action Plan (ENAP) to end preventable neonatal deaths, decrease disability, and end preventable stillbirths by 2030 [36] is an ambitious goal and the scientific review of perinatal deaths to explore causes and generate appropriate action plans will be very important to accomplish the goal. The present research with added efforts to generate such evidence in addition to the ongoing MPDSR system can be a useful step and can be a milestone so that the appropriate investigations to generate evidence on the causes of death and preventability can be effectively utilized. It can guide the policymakers to strengthen the DSS across different contexts globally.

The results of MITS have been validated with CDA and has been demonstrated to produce good agreement in determining the cause of death across the age groups [37, 38] and MITS can be a useful alternative in preterm deaths [38]. Initial findings from the CHAMPS study have shown that CoD using MITS enhanced the determination of causes of death in stillbirths, neonatal deaths, and under-5 deaths [15]. The causes of stillbirths and preterm neonatal deaths were also determined exhaustively based on the utilization of MITS in the PURPOSe study [39, 40]. The number of tissues and specimens sampled and the laboratory examinations performed are exhaustive in those projects, and the utilization of the tests to effectively determine the cause of death has to be meticulously analyzed in order to advocate for their application in routine cases, particularly to make the investigation cost-effective and for sustainability. Based on the latest evidence and observations, Goldenberg et al. [41] have recommended the use of clinical history, evaluation of the placenta, fetal external examination, and fetal tissues obtained by MITS, particularly the lungs for histology and microbiology, brain/cerebral spinal fluid (CSF) and fetal blood for microbiological analysis. In this project, we aim to generate further evidence to advocate for the determination of perinatal deaths so that MITS can be effectively integrated into the ongoing DSS in the global context.

### Potential biases

There may be recall bias from the respondents in community perinatal deaths, which can be minimized by visiting the households soon after the mourning. This can be achieved by effective mobilization of FCHVs, community leaders, and nurses and getting the notification of the deaths on time in order to perform the VA soon after the death.

### Limitation of the study

The nearest family members may be reluctant to provide complete history of the decedent. We will try to overcome this issue by employing trained grief counsellors to counsel the relatives before the history is taken. The MITS technique may miss out some pathologies and anomalies present in the organs. So, it may not yield same results as that by CDA.

## Data Availability

No datasets were generated or analysed during the current study.

## Abbreviations

ANC: Absolute Neutrophil Count
BHI: Brain Heart Infusion
CDA: Complete Diagnostic Autopsy
CHAMPS: Child Health and Mortality Prevention Surveillance
CoD: Cause of Death
CRF: Case Report Forms
CSF: Cerebrospinal Fluid
DeCoDe: Determination of the Cause of Death
DLC: Differential Leukocyte Count
DSS: Death Surveillance System
EDTA: Ethylenediamine Tetra Acetic Acid
ESR: Erythrocyte Sedimentation Rate
FCHVs: Female Community Health Volunteers
H&E: Hematoxylin and Eosin
HbsAg: Hepatitis B surface Antigen
HIV: Human Immunodeficiency Virus
ICD PM: International Classification of Diseases Perinatal Mortality
IT ratio: Immature to Total cell ratio
LMP: Last Menstrual Period
LMICs: Low- and Middle-Income Countries
MITS: Minimally Invasive Tissue Sampling
MPDSR: Maternal and Perinatal Death Surveillance and Response
PCR: Polymerase Chain Reaction
PDR: Perinatal Death Review
PURPOSe: The Project to Understand and Research Preterm Pregnancy Outcomes and Stillbirths in South Asia
RT-PCR: Real Time Polymerase Chain Reaction
TORCH: Toxoplasmosis, Rubella Cytomegalovirus and Herpes Simplex Virus 2
VA: Verbal Autopsy
VERS: Vital Event Registration System
VTM: Viral Transfer Media
WHO: World Health Organization
